# Socioeconomic inequalities in avoidable mortality in Italy: results from a nationwide longitudinal cohort

**DOI:** 10.1186/s12889-024-18205-6

**Published:** 2024-03-11

**Authors:** Alessio Petrelli, Martina Ventura, Anteo Di Napoli, Marilena Pappagallo, Silvia Simeoni, Luisa Frova

**Affiliations:** 1 National Institute for Health, Migration and Poverty (INMP), Via di San Gallicano, 25/a, 00153 Rome, Italy; 2 National Institute of Statistics (Istat), Viale Liegi 13, 00198 Rome, Italy

**Keywords:** Avoidable mortality, Preventable mortality, Treatable mortality, Socioeconomic inequalities, Education level

## Abstract

**Background:**

Disparities in avoidable mortality have never been evaluated in Italy at the national level. The present study aimed to assess the association between socioeconomic status and avoidable mortality.

**Methods:**

The nationwide closed cohort of the 2011 Census of Population and Housing was followed up for 2012–2019 mortality. Outcomes of preventable and of treatable mortality were separately evaluated among people aged 30–74. Education level (elementary school or less, middle school, high school diploma, university degree or more) and residence macro area (North-West, North-East, Center, South-Islands) were the exposures, for which adjusted mortality rate ratios (MRRs) were calculated through multivariate quasi-Poisson regression models, adjusted for age at death. Relative index of inequalities was estimated for preventable, treatable, and non-avoidable mortality and for some specific causes.

**Results:**

The cohort consisted of 35,708,459 residents (48.8% men, 17.5% aged 65–74), 34% with a high school diploma, 33.5% living in the South-Islands; 1,127,760 deaths were observed, of which 65.2% for avoidable causes (40.4% preventable and 24.9% treatable). Inverse trends between education level and mortality were observed for all causes; comparing the least with the most educated groups, a strong association was observed for preventable (males MRR = 2.39; females MRR = 1.65) and for treatable causes of death (males MRR = 1.93; females MRR = 1.45). The greatest inequalities were observed for HIV/AIDS and alcohol-related diseases (both sexes), drug-related diseases and tuberculosis (males), and diabetes mellitus, cardiovascular diseases, and renal failure (females). Excess risk of preventable and of treatable mortality were observed for the South-Islands.

**Conclusions:**

Socioeconomic inequalities in mortality persist in Italy, with an extremely varied response to policies at the regional level, representing a possible missed gain in health and suggesting a reassessment of priorities and definition of health targets.

**Supplementary Information:**

The online version contains supplementary material available at 10.1186/s12889-024-18205-6.

## Background

Wherever data are available, mortality rates are higher among people in disadvantaged socioeconomic positions [1], regardless of the country’s level of development. These differences are also present in more developed countries with advanced health-care systems and complex social welfare systems [2, 3]. Disparities in mortality rates due to socioeconomic differences have been observed throughout the 20th century, despite massive changes in disease patterns and their determinants [4].

Many conceptual models have been developed to explain socioeconomic inequalities in health. According to the theory of the “fundamental cause” of inequalities in mortality, the differences in individuals’ social positions is determined by their control of and capacity to use resources such as money, knowledge, prestige, power, and beneficial social connections, which gives them control over life and circumstances. It is hypothesized that such resources drive an individual’s health by influencing choices regarding healthy lifestyles and psychosocial-related factors such as career path and life conditions, which can specifically contribute to stressor mechanisms. Clearly, these resources can also influence an individual’s ability to access appropriate preventive interventions or health care, all pathogenic mechanism [5, 6]. Analogous mechanisms may be in action at the community level; for example, differences in social capital can lead to limitations in accessing infrastructural resources useful for health, and more unhealthy living conditions can expose populations to higher environmental exposures.

This interpretation of inequalities in mortality refers to what has been called a “meta-mechanism,” an overarching mechanism that explains how multiple specific mechanisms reproduce a particular relationship in different places and at different times. What’s more, this mechanism is not in conflict with the approach based on specific determinants such as the higher prevalence of unfavorable material, psychosocial and behavioral factors in lower socioeconomic groups [7].

On the other hand, health systems play a role in contributing to equity in health by ensuring that the entire population receives both appropriate and timely health treatments and access to prevention strategies and interventions aimed at effectively controlling risk factors.

Reducing socioeconomic health inequalities represents a major goal of the health policy in most countries, including Italy, where it is one of the pillars of the National Plan of Prevention [8] (https://www.salute.gov.it/portale/news/p3_2_1_1_1.jsp?id=5029&menu=notizie).

Despite universal health coverage in Italy, differences in health outcomes due to socioeconomic inequalities persist, albeit with less intensity when compared with most European countries both in terms of general mortality [2] and of premature mortality [9]. In Italy, males with a lower education level show a 3-year shorter life expectancy at birth than those with a higher education level; residents in southern Italy have a life expectancy that is 1 year less than that in the northern and central regions, regardless of education level. The same pattern has been observed for females, although less marked [10].

The concept of avoidable mortality, developed during the 1970s, has been revised many times since then according to different contexts or purposes [11]. In the most recent approach, developed by the Organisation for Economic Co-operation and Development (OECD), avoidable causes of death are those which, in the light of medical knowledge, technology, and the determinants of health at the time of death, could have been avoided through timely and effective health-care interventions (treatable or amenable mortality), including secondary prevention and treatment (i.e., after the onset of disease, to reduce case fatality) or effective public health and primary prevention (i.e., before the onset of disease/injury, to reduce incidence) (preventable mortality). Despite limitations due to the heterogeneity in the causes of death considered and to the lack of a univocal definition and identification of exactly what is being measured [12], avoidable mortality remains a useful measure to evaluate the impact of health-care systems on the health of populations.

Avoidable mortality has constantly declined in the European Union over the last decades. In 2019, about 1.2 million deaths among people aged less than 75 years (equivalent to 24.3 deaths per 10,000 inhabitants) could have been avoided in the EU, either through better health-care systems and/or better public health interventions [13].

Italy is one of the European countries with the lowest avoidable mortality rate, which decreased from 19.7*10,000 inhabitants in 2012 to 16.5 in 2019 [13] (https://ec.europa.eu/eurostat/databrowser/view/hlth_cd_apr/default/table?lang=en). The reduction involved primarily the preventable causes of death, in particular lung cancer and ischemic heart disease, especially among males [14]. Socioeconomic gradients in premature mortality have been well documented, with rates higher for the most disadvantaged groups and lower for the least disadvantaged groups [9]. A recent large English study estimated that socioeconomic inequalities explain one-third of premature mortality [15].

In Italy, although socioeconomic inequalities in health are well documented, disparities in avoidable mortality at the national level have not been investigated.

To date, international studies involving Italy or conducted in Italy have considered some metropolitan areas, such as Turin or some cities in Tuscany [16].

In Italy, a national follow-up system based on the cohort generated by the record linkage between the Italian Register of Causes of Death and the Population and Housing Census archives has been developed by the Italian National Institute of Statistics [17]. This follow-up system makes it possible to explore demographic and socioeconomic inequalities longitudinally and for the entire Italian population in general and for cause-specific mortality.

For the purpose of our study, we used education level as a pragmatic measure of socioeconomic status, which is reasonably comparable across different contexts [18]. Education rarely changes over time and so is less sensitive to reverse causation for adults, as it does not change if one’s health deteriorates. It is therefore particularly appropriate in studies exploring mortality inequalities. Higher education is related to health through numerous pathways, such as a smaller risk of unemployment, higher income, good housing conditions, lower levels of unhealthy behavior, lower exposure to psychosocial risk factors, and better social support [19]. Education level is associated with the types of resources (e.g., greater health awareness and health literacy [20]) that are required to improve health generally but that may also be key to navigating the health-care system, thereby reducing the risk of amenable death specifically.

Moreover, in Italy, a North-South gradient in socioeconomic conditions and health persists across the country, with a higher concentration of disadvantaged people and worse living conditions in the southern regions compared to the rest of Italy, leading to higher risk of mortality for many causes of death [10].

The present study aimed to assess the association between socioeconomic status and avoidable mortality, considering both preventable or and treatable mortality.

## Methods

The study was conducted on the population cohort conceived within the project “Socioeconomic differences in mortality”, part of the National Statistical Program (PSN) approved by the Italian Data Protection Authority (IF IST 2646) and was approved by the Director of the Central Directorate for Social Statistics and Population Census of the National Institute of Statistics (Istat) and by the General Director of the National Institute for Health, Migration and Poverty (INMP). This is a population-based national closed cohort of all residents recorded in the 2011 Census of Population and Housing (2011), with follow-up data for mortality from January 2012 to December 2019. Information on death was taken from the Italian Register of Causes of Death (IRCoD) Register. Moreover, the Resident Population Register (RPR) was used to collect individual data on demographic events occurring in Italy or abroad among the resident population to take into account any migration events and their relative date.

Using a retrospective longitudinal design, subjects entered the cohort on 1 January 2012. They were followed up until death, emigration, or the last available year of mortality data (2019), whichever came first, yielding a maximum of 8 years of follow-up. Mortality data were obtained through a deterministic record linkage with the IRCoD by using individuals’ fiscal code (a unique personal identification number issued to all residents in Italy at birth or upon immigration) as a linkage key. The reliability of the fiscal code was very high in all the registers, making it possible to link 97.1% of all deaths among the Census population occurring in Italy.

The databases used were created and managed by the Italian National Institute of Statistics, which checked for duplicates before their final release.

For the purpose of this study, the population aged 30–74 years was considered. The choice of the lower age limit was motivated by individuals’ having reached a sufficiently established education level, while the upper age limit of 74 was chosen because it is the general age threshold traditionally used in avoidable mortality lists in developed countries. This age threshold still reflects the life expectancy at birth in those OECD and EU countries with the lowest life expectancies.

Avoidable causes of death were divided into preventable and treatable according to the 2019 OECD- Eurostat classification. In 2019, the OECD and Eurostat worked with an expert group to develop new joint lists of preventable and treatable causes of mortality. These lists were built on earlier work by researchers (e.g., Nolte and McKee, 2004 and 2011), by some OECD countries, and by Eurostat. The new OECD-Eurostat lists were approved during the OECD Working Party on Health Statistics meeting in October 2018 and during the Eurostat Working Group on Public Health Statistics in December 2018 [21].

Consistently with the OECD-Eurostat classification, mortality in this study was analyzed separately as:

1. preventable mortality: refers to the causes of death that can be mainly avoided through effective public health and primary prevention interventions (i.e., before the onset of disease/injury, to reduce incidence);
2. treatable mortality: refers to the causes of death that can be mainly avoided through timely and effective health-care interventions, including secondary prevention and treatment (i.e., after the onset of disease, to reduce case fatality).

Some causes of deaths are both preventable and treatable; in these cases, deaths are equally assigned to both the preventable and the treatable groups.

For the purpose of the study, the remaining causes of death, not included in the aforementioned groups, were considered together and analyzed as “non-avoidable” mortality.

In this study, specific causes or subgroups of causes of death were also selected for relevance and analyzed to better explore the effect of socioeconomic status on preventable and treatable mortality. The complete list of the causes considered is shown in Table 1.

**Table 1:** Lists of preventable and treatable causes of death, selected and grouped for this study

Cause	ICD10 codes	preventable mortality	treatable mortality
HIV/AIDS	B20-B24	x	
Tuberculosis	A15-A19, B90, J65	x (50%)	x (50%)
Neoplasms	C00-C16, C22, C33-C34, C43, C45, C53, C67	x	
	C18-C21, C50, C53-C55, C62, C73,C81, C91.0, C91.1, D10-D36		x
Lip, oral cavity, pharynx and esophageal cancers	C00-C15	x	
Stomach cancer	C16	x	
Liver cancer	C22	x	
Lung cancer	C33-C34	x	
Colorectal cancer	C18-C21		x
Cervical cancer	C53	x (50%)	x (50%)
Breast cancer	C50		x
Uterine cancer	C54,C55		x
Hodgkin’s disease + Lymphoid leukaemia	C81,C91.0, C91.1		x
Diabetes mellitus	E10-E14	x (50%)	x (50%)
Hypertensive diseases	I10-I13, I15	x (50%)	x (50%)
Ischemic heart diseases	I20-I25	x (50%)	x (50%)
Cerebrovascular diseases	I60-I69	x (50%)	x (50%)
Diseases of the respiratory system	J09-J11, J13-J14, J40-J44, J60-J64, J66-J70, J82, J92	x	
	J00-J06, J12, J15-J18, J20-J22, J30-J39, J45-J47, J80-J81, J85, J86, J90, J93, J94		x
Diseases of the digestive system	K25-K28, K35-K38, K40-K46, K80-K83, K85.0,K85.1,K85.3,K85.8,K85.9, K86.1,K86.2,K86.3,K86.8,K86.9		x
Diseases of the genitourinary system	N00-N07, N13,N20-N21, N23, N25-N27, N35, N40, N34.1, N70-N73, N75.0,N75.1,N76.4,N76.6		x
Renal failure	N17-N19		x
Transport accidents	V01-V99	x	
Alcohol-related deaths	E24.4, F10, G31.2, G62.1, G72.1, I42.6, K29.2, K70, K85.2, K86.0, Q86.0, R78.0, X45, X65, Y15	x	
Drug-related deaths	F11-F16, F18-F19, X40-X44, X85, Y10-Y14, X60-X64, K73, K74.0-K74.2, K74.6	x	
Other avoidable		x	
			x

*Source: OECD Eurostat (2019)*

Education level was considered as the main exposure, as a proxy indicator of socioeconomic status, and was classified into four categories:

- elementary school or less (up to 5 years of schooling).
- middle school (8 years).
- high school diploma (13 years).
- university degree or more (≥ 16 years).

The residence area was considered as another potential socioeconomic factor affecting mortality; the 20 Italian regions were aggregated in 4 geographic macro areas: North-West (Piedmont, Valle d’Aosta, Lombardy, Liguria), North-East (Trentino Alto Adige, Veneto, Friuli Venetia Giulia, Emilia-Romagna), Center (Tuscany, Umbria, Marche, Latium), and South and Islands (Abruzzi, Molise, Campania, Apulia, Basilicata, Calabria, Sicily, Sardinia).

### Statistical analysis

Baseline sociodemographic characteristics of the cohort and the deaths occurring during follow-up are described for males and females.

Crude and age-standardized mortality rates (ASMR) per 100,000 person-years for education level and geographic macro area of residence were calculated separately by group of causes (preventable, treatable, and non-avoidable) and sex. The direct standardization was computed using as weights the 2013 European Standard Population [22].

To evaluate the effect of education level on mortality, multivariate quasi-Poisson regression models for overdispersed count data with log link function [23] were performed by sex and group of causes, taking into account age at death and macro area of residence. We also estimated models not adjusted for area of residence; as the results overlapped, the data are not shown in the tables. Adjusted mortality rate ratios (MRRs) with 95% confidence intervals (CIs) were estimated, using “university degree or more” as the reference category.

The educational gradient was also evaluated through the relative index of inequalities (RII) [24], adjusted for age and macro area of residence, which was estimated considering mortality for the main groups of causes (preventable, treatable, and non-avoidable) and for the cause-specific mortality as defined in Table 1. In addition, an in-depth assessment was done on RIIs, stratifying the study population by age group (30–59; 60–74 years).

Finally, geographic differences in mortality were evaluated through MRRs, adjusted for age and education level, by group of causes (preventable, treatable, and non-avoidable) and sex.

All methods were carried out in accordance with relevant guidelines and regulations. All analyses were performed using SAS® System version 9.4.

## Results

Table 2 shows the distribution of the cohort, person-years, and deaths for all the variables considered in the analyses. The study population consisted of 35,708,459 people resident in Italy, 48.8% of whom were males and 17.5% were aged 65–74; high school diploma was the most frequent education level (34%), and the South and Islands was the geographic macro area where the highest proportion of people lived (33.5%). During the study period (2012–2019), 1,127,760 deaths were observed, mostly among males (62%), people aged 65–74 (73%), those with an education level of elementary school or less (38%), and in the South and Islands (36%).

**Table 2:** Baseline characteristics of the study population by sex, number of deaths, and person-years between 2012 and 2019

	MALES						FEMALES						TOTAL					
	Cohort		Person-years		Deaths		Cohort		Person-years		Deaths		Cohort		Person-years		Deaths	
	N	%	N	%	N	%	N	%	N	%	N	%	N	%	N	%	N	%
Total*	17,414,659	*48.8*	112,674,342	*48.8*	699,340	*62.0*	18,293,800	*51.2*	118,405,167	*51.2*	428,420	*38.0*	35,708,459	*100.0*	231,079,508	*100.0*	1,127,760	*100.0*
**Characteristics**																		
**Age at death**																		
30–44	6,488,947	*37.3*	33,461,071	*29.7*	30,307	*4.3*	6,563,785	*35.9*	33,876,334	*28.6*	18,246	*4.3*	13,052,732	*36.6*	67,337,405	*29.1*	48,553	*4.3*
45–64	6,211,074	*35.7*	45,662,747	*40.5*	157,096	*22.5*	6,472,528	*35.4*	47,364,814	*40.0*	98,514	*23.0*	16,404,059	*45.9*	93,027,561	*40.3*	255,610	*22.7*
65–74	4,714,638	*27.1*	33,550,523	*29.8*	511,937	*73.2*	5,257,487	*28.7*	37,164,018	*31.4*	311,660	*72.7*	6,251,668	*17.5*	70,714,542	*30.6*	823,597	*73.0*
**Education level**																		
Elementary school or less	2,869,033	*16.5*	15,726,148	*14.0*	242,927	*34.7*	4,140,476	*22.6*	22,937,495	*19.4*	185,710	*43.3*	7,009,509	*19.6*	38,663,643	*16.7*	428,637	*38.0*
Middle school	6,215,192	*35.7*	41,105,432	*36.5*	242,853	*34.7*	5,366,642	*29.3*	35,800,194	*30.2*	117,649	*27.5*	11,581,834	*32.4*	76,905,626	*33.3*	360,502	*32.0*
High school diploma	6,048,594	*34.7*	40,632,855	*36.1*	164,688	*23.5*	6,086,720	*33.3*	41,268,661	*34.9*	93,142	*21.7*	12,135,314	*34.0*	81,901,516	*35.4*	257,830	*22.9*
University degree or more	2,281,829	*13.1*	15,209,830	*13.5*	48,872	*7.0*	2,699,959	*14.8*	18,398,795	*15.5*	31,919	*7.5*	4,981,788	*14.0*	33,608,625	*14.5*	80,791	*7.2*
**Macro area of residence**																		
North-West	4,757,998	*27.3*	30,657,397	*27.2*	186,412	*26.7*	4,934,818	*27.0*	31,757,474	*26.8*	113,538	*26.5*	9,692,816	*27.1*	62,414,871	*27.0*	299,950	*26.6*
North-East	3,450,725	*19.8*	22,309,764	*19.8*	129,647	*18.5*	3,548,493	*19.4*	22,934,444	*19.4*	77,775	*18.2*	6,999,218	*19.6*	45,244,208	*19.6*	207,422	*18.4*
Center	3,410,619	*19.6*	22,030,981	*19.6*	130,869	*18.7*	3,656,430	*20.0*	23,619,503	*19.9*	83,312	*19.4*	7,067,049	*19.8*	45,650,484	*19.8*	214,181	*19.0*
South and Islands	5,795,317	*33.3*	37,676,200	*33.4*	252,412	*36.1*	6,154,059	*33.6*	40,093,746	*33.9*	153,795	*35.9*	11,949,376	*33.5*	77,769,946	*33.7*	406,207	*36.0*

* Total percentages by sex

As described in Tables 3 and 65.2% of all deaths were due to avoidable causes, of which 40.4% and 24.9% to preventable and treatable causes, respectively. The distribution of deaths by group of causes was different by sex, with the highest proportion of deaths for preventable causes in males and for non-avoidable causes in females. Considering the ASMRs, an inverse trend of mortality by education level was observed for preventable, treatable, and non-avoidable causes of death for both males and females. However, rates and inequalities were systematically higher among males for all groups of causes of death, particularly for preventable causes of death. Regarding the geographic macro area of residence, significantly higher ASMRs of mortality were observed in the South and Islands for all groups of causes of death.

**Table 3:** Distribution of deaths and age-standardized mortality rates (ASMR) for education level and macro area of residence, by group of causes and sex

	PREVENTABLE				TREATABLE				NON-AVOIDABLE			
	Deaths		ASMR *100.000 py	95%CI	Deaths		ASMR *100.000 py	95%CI	Deaths		ASMR *100.000 py	95%CI
	N	%			N	%			N	%		
**TOTAL**	**455,121**	***40.4***			**280,437**	***24.9***			**392,203**	***34.8***		
**MALES**	**324,081**	***46.3***			**142,714**	***20.4***			**232,545**	***33.3***		
**Education level**												
Elementary school or less	114,202	*35.2*	38.69	38.38-39.00	51,169	*35.9*	16.50	16.30-16.69	77,556	*33.4*	27.00	26.73–27.28
Middle school	116,393	*35.9*	29.75	29.57–29.93	47,733	*33.4*	12.47	12.36–12.59	78,727	*33.9*	20.37	20.22–20.51
High school diploma	73,280	*22.6*	22.13	21.97–22.30	33,592	*23.5*	10.43	10.31–10.55	57,816	*24.9*	17.68	17.53–17.83
University degree or more	20,206	*6.2*	15.86	15.64–16.09	10,220	*7.2*	8.17	8.01–8.33	18,446	*7.9*	14.57	14.35–14.79
**Macro area of residence**				*-*		0.0		*-*				*-*
North-West	87,827	*27.1*	27.10	26.92–27.28	36,517	*25.6*	11.26	11.14–11.37	62,068	*26.7*	19.12	18.96–19.27
North-East	59,574	*18.4*	25.68	25.47–25.88	25,266	*17.7*	10.89	10.76–11.03	44,807	*19.3*	19.30	19.12–19.48
Center	60,396	*18.6*	26.08	25.87–26.29	27,332	*19.2*	11.77	11.63–11.91	43,142	*18.6*	18.60	18.42–18.78
South and Islands	116,285	*35.9*	30.14	29.96–30.31	53,600	*37.6*	13.92	13.80-14.04	82,528	*35.5*	21.36	21.22–21.51
**FEMALES**	**131,040**	***30.6***			**137,723**	***32.1***			**159,658**	***37.3***		
**Education level**												
Elementary school or less	57,344	*43.8*	12.50	12.34–12.67	58,242	*42.3*	13.23	13.06–13.41	70,124	*43.9*	15.88	15.67–16.08
Middle school	36,786	*28.1*	10.51	10.40-10.62	37,412	*27.2*	10.56	10.45–10.67	43,451	*27.2*	12.54	12.42–12.66
High school diploma	27,829	*21.2*	8.96	8.84–9.07	31,088	*22.6*	9.56	9.45–9.68	34,226	*21.4*	11.11	10.99–11.24
University degree or more	9,081	*6.9*	7.43	7.26–7.60	10,981	*8.0*	8.56	8.39–8.74	11,857	*7.4*	9.83	9.63–10.02
**Macro area of residence**				*-*				*-*				*-*
North-West	35,504	*27.1*	10.02	9.92–10.13	35,766	*26.0*	10.08	9.97–10.18	42,269	*26.5*	11.89	11.78–12.01
North-East	24,172	*18.4*	9.63	9.51–9.76	23,795	*17.3*	9.45	9.33–9.58	29,809	*18.7*	11.85	11.72–11.99
Center	26,258	*20.0*	10.11	9.99–10.23	26,204	*19.0*	10.08	9.96–10.20	30,850	*19.3*	11.86	11.73-12.00
South and Islands	45,107	*34.4*	10.61	10.51–10.71	51,959	*37.7*	12.18	12.07–12.28	56,730	*35.5*	13.34	13.23–13.45

**Fig. 1:**
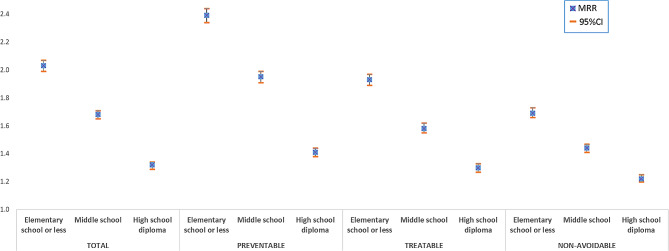
Mortality rate ratios (MRRs) from multivariate Poisson regression model, by education level, adjusted by age and macro area of residence (reference category “University degree or more”) - Males

**Fig. 2:**
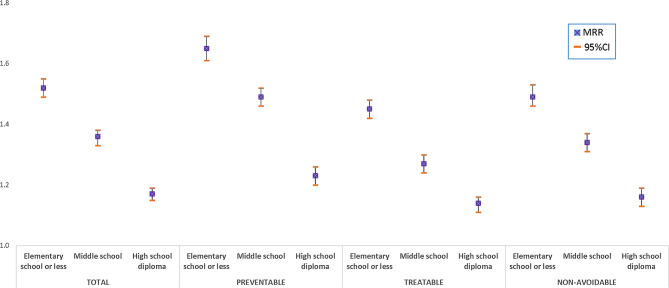
Mortality rate ratios (MRRs) from multivariate Poisson regression model, by education level, adjusted by age and macro area of residence (reference category “University degree or more”) - Females

Figures 1 and 2 show the results of multivariate statistical models for males and females, respectively. Among males, a statistically significant inverse trend between mortality and education level was observed for all the groups of causes and for overall mortality, used as a comparison. Mortality among the less educated was about double compared to that of the most educated (MRR = 2.03; 95%CI: 1.99–2.07), with a stronger association between education level and mortality observed for preventable causes of death (MRR = 2.39; 95%CI: 2.34–2.44) than for treatable causes of death (MRR = 1.93; 95%CI: 1.89–1.97). The findings for females showed a similar trend, although the inequalities are less marked. MRR for elementary school or less compared with university degree or more was 1.52 (95%CI: 1.49–1.55), 1.65 (95%CI: 1.61–1.69) and 1.45 (95%CI: 1.42–1.48) for total, preventable, and treatable mortality, respectively.

Table 4 shows the RII for education level by groups of causes and for the selected specific causes of death. Among males, RII was 2.18 for all-cause mortality, 2.61 for preventable mortality, and 2.04 for treatable mortality. The highest inequalities were observed for HIV/AIDS (RII = 6.31), alcohol-related (RII = 5.76), tuberculosis (RII = 5.30), and drug-related (RII = 4.81) deaths. Moreover, we found that inequalities were more marked among the preventable diseases of the respiratory system, lip, oral cavity, pharynx, and esophageal cancers, diabetes mellitus, and renal failure.

**Table 4:** Relative index of inequalities (RII) for education level by cause of death, adjusted for age and macro area of residence

Causes	MALES			FEMALES		
	N	RII	95%CI	N	RII	95%CI
All Preventable causes	324,081	2.61	2.56–2.66	131,040	1.73	1.69–1.76
All Amenable causes	142,714	2.04	2.00-2.08	137,723	1.52	1.49–1.55
Non-avoidable	232,545	1.79	1.75–1.82	159,658	1.57	1.53–1.61
HIV/AIDS	2,728	6.31	5.76–6.92	719	7.30	6.49–8.21
Tuberculosis	349	5.30	4.71–5.95	176	2.41	2.13–2.72
All PREVENTABLE cancers	167,423	2.52	2.47–2.56	67,367	1.31	1.28–1.34
All TREATABLE cancers	38,015	1.44	1.40–1.48	82,811	1.04	1.01–1.06
Lip, oral cavity, pharynx, and esophageal cancers	15,713	3.17	3.03–3.31	4,634	1.52	1.43–1.61
Stomach cancer	18,399	2.56	2.48–2.65	9,710	2.26	2.16–2.36
Liver cancer	24,313	2.92	2.81–3.02	7,765	2.08	1.98–2.18
Lung cancer	89,366	2.59	2.54–2.64	36,361	1.03	1.00-1.06
Colorectal cancer	32,345	1.45	1.41–1.49	21,742	1.21	1.17–1.25
Cervical cancer				2,163	2.19	2.02–2.36
Breast cancer				45,877	0.85	0.83–0.88
Uterine cancer				9,349	1.48	1.42–1.55
Hodgkin’s disease + Lymphoid leukaemia	3,075	1.38	1.30–1.46	1,793	1.07	1.00-1.15
Diabetes mellitus	21,600	3.11	2.99–3.23	12,338	5.21	4.94–5.48
Hypertensive diseases	14,605	1.98	1.91–2.06	8,805	2.62	2.50–2.74
Ischaemic heart diseases	73,619	1.98	1.94–2.03	23,102	2.68	2.58–2.78
Cerebrovascular diseases	30,661	2.29	2.22–2.37	21,168	2.17	2.09–2.25
All PREVENTABLE diseases of the respiratory system	16,154	4.86	4.64–5.08	7,818	2.39	2.26–2.52
All TREATABLE diseases of the respiratory system	8,203	3.00	2.84–3.17	4,715	3.08	2.89–3.30
All TREATABLE diseases of the digestive system	4,181	2.66	2.51–2.83	2,405	3.32	3.09–3.57
Diseases of the genitourinary system (except renal failure)	545	1.62	1.48–1.76	334	3.24	2.89–3.63
Renal failure	4,982	3.04	2.86–3.23	3,353	5.40	5.00-5.82
Transport accidents	10,773	2.40	2.30–2.52	2,653	1.39	1.29–1.49
Alcohol-related deaths	17,383	5.76	5.46–6.08	6,491	4.13	3.88–4.40
Drug-related deaths	1,669	4.81	4.34–5.33	646	1.21	1.09–1.34
All causes	699,340	2.18	2.14–2.22	428,420	1.60	1.57–1.63

Among females, RII resulted 1.60 for all-cause mortality and 1.73 and 1.52 for preventable and treatable mortality, respectively. Compared with males, higher inequalities were found in females for HIV/AIDS (RII = 7.30), diabetes mellitus (RII = 5.21), hypertensive diseases (RII = 2.62), ischemic heart disease (RII = 2.68), treatable diseases of the digestive system (RII = 3.32), and diseases of the genitourinary system (RII = 3.24), including renal failure (RII = 5.40). In addition, inequalities among females were particularly higher for alcohol-related diseases (RII = 4.13). Lastly, an inverse association was found for breast cancer, with an RII of 0.85, indicating a higher risk for females with higher education.

The age-adjusted estimates of mortality risks by geographic macro area (Supplementary Table 1) show a statistically significant excess of risk for the South and Islands when compared with the North-West for overall mortality and for both preventable and treatable mortality. In particular, this excess was more pronounced for treatable mortality among both males (MRR = 1.21) and females (MRR = 1.18). Instead, the comparison with other geographic macro areas did not show statistically significant differences in risk.

## Discussion

The results of our study show an inverse socioeconomic gradient in avoidable mortality in Italy, both for preventable and for treatable mortality. The association between education level and mortality was stronger for preventable mortality compared to treatable mortality and for avoidable compared to non-avoidable mortality.

The analysis of causes of death showed the highest inequalities for HIV/AIDS and alcohol-related diseases in both sexes, for drug-related diseases and tuberculosis among males, and for diabetes mellitus, cardiovascular diseases, and renal failure among females.

This is the first study to analyze avoidable mortality in Italy with coverage of the entire Italian population. Compared to previous studies in which Italy was represented by the city of Turin or some cities in Tuscany, the inequalities measured nationally appear to be stronger for treatable mortality among both males and females [25].

Since the Italian National Health Service guarantees universal access to the entire population for all health services, one would expect that social disparities in health outcomes would be very limited. Our findings indicate that socioeconomic inequalities in preventable mortality are wider than corresponding inequalities in overall mortality. These results are consistent with the conceptual model of the “fundamental causes of death,” since the unequal distribution of resources by socioeconomic level theorized by the model would be more accentuated precisely for those causes of death for which greater advantage can be obtained in terms of prevention. Our results are consistent with those found in other European countries [9], including those countries with universal health care and those which invest substantial economic resources in their welfare systems such as the Scandinavian countries [26–29].

Moreover, in Italy, socioeconomic inequalities in treatable mortality appear to be lower compared to the majority of European countries. Indeed, as a recent international paper documented, the relative inequalities in treatable mortality in Italy were only higher than those observed in Austria, Belgium, Denmark, Scotland, and Spain but lower than in the majority of European countries [20, 29]. Also, there appeared to be fewer inequalities in preventable mortality in Italy than in other developed countries [30].

Many factors have been evoked to explain the relative advantage in mortality inequalities in Italy, which aims to guarantee equally-distributed protection across Italian society. These factors include the Mediterranean diet, a heritage shared by the various social strata, the universal health care system, which provides free health care to all through the Italian National Health Service, and the protective network of the family, which is still strong in Italy and which compensates for any insufficiencies in services, especially in the care of the elderly and the disabled. However, it should be pointed out that both proper nutrition and the family network are assets that are undergoing a marked deterioration. For example, the increasing proportion of obese and overweight individuals, which has reached 44.7% [31], is a sign of a change in eating habits, and the serious situation of families with a disabled family member bears witness to the lack of services. Finally, the potential socioeconomic lags in the diffusion of new technologies (e.g., in medical care) [32] and interventions (e.g., in health promotion) [33, 34] should not be underestimated.

Our in-depth assessment of RIIs by age group (Supplementary Table 2) highlights that social inequalities in mortality are less pronounced for the older age groups, probably due to a mix of factors: they have greater welfare protection and, although perhaps less relevant to our cohort, because the vulnerable have already suffered a disadvantage in terms of premature mortality, as the poor tend to fall ill and die at a younger age.

The higher risk of avoidable mortality among the less educated is confirmed for almost all the groups of causes of death analyzed. For all causes of death, the disadvantage in males is stronger in the less educated, although the magnitude of excess mortality varies according to the cause of death considered. Diseases that are strongly associated with risk behavior and for which it is easy to identify risk factors on which to intervene are those with the greatest social disparities: AIDS, associated with drug abuse and unprotected sex; liver cancers, associated with alcohol abuse; cancers of the upper digestive tract (UGI) among males, associated with smoking and alcohol abuse; stomach cancers, associated with infections and poor food hygiene; transport accidents among males, related to road safety; respiratory system diseases among males, associated with work-related risks and smoking; diabetes mellitus, especially among females, associated with obesity; lung cancer among males, related to smoking.

The role of prevention is particularly relevant in terms of cardiovascular disease (CVD)-related mortality, which could largely be prevented by eliminating smoking, improving diet, reducing alcohol intake, and increasing physical activity [10]. In fact, we observed an overlap of the geographic patterns of risk factors and cardiovascular mortality distribution, with higher CVD-related mortality in the southern regions of Italy, regardless of social status [10].

Those diseases associated with alcohol abuse or with the hepatitis viruses (liver cancer or cirrhosis), with smoking and occupational risks (lung cancers, upper respiratory and digestive tract cancers), or safety (accidents) showed a geographic pattern, confirming as a priority for the Italian National Prevention Plan the reduction in the inequalities in risk factors. The findings of lower mortality rates for malignant neoplasms of the colon are partially explained by the protective effect of screening when implemented early and effectively [10].

Socioeconomic inequalities in avoidable mortality were less pronounced among females than in males. This difference could be partially due to the fact that the only causes of death for which an inverse relationship with education was not observed concerned females, namely cancers with a high lethality rate: lung cancer, for which no difference by education level was observed, and breast cancer, for which the most educated are at higher risk.

More educated women adopt a risk factor such as smoking earlier than those less educated and postpone pregnancy (Mac Dorman, 2021), leading to a reduction in their health advantage over the less educated. This effect also leads to an increase in the target population for tobacco prevention.

Finally, social disadvantage linked to low education level also acted heterogeneously in the different geographic macro areas, determining higher mortality risk mainly in the South. Our study confirms the recent observation [10] that in southern Italy, the effect of education level plus contextual factors determines a further systematic disadvantage in mortality.

Our findings show a higher risk of death for those living in the South and Islands for all groups of causes, particularly for causes due to treatable conditions (an excess of more than 20% for both sexes). Differences among geographic areas in Italy can largely be attributed to the Italian NHS’s ability to offer screening programs that reach the target population. It is well known that organized screening programs can reduce inequalities, as access to opportunistic screening is more probable among affluent people [35, 36]. Breast cancer mortality is decreasing faster in the northern and central regions than in the southern regions of Italy, a trend partly explained by the trends in mammography screening coverage by geographic area, which is particularly evident in the age range targeted by breast cancer screening [37].

### Strengths

One of the main strengths of our study is that it is based on the whole population of residents in Italy, making it possible to provide a detailed picture of the phenomenon.

This nationwide data source allows a comprehensive assessment of socioeconomic and geographic inequalities in mortality, including evaluations of specific causes of death.

### Limitations

One of the main limitations of the study hinges on the definition of the indicators of avoidable mortality; consequently, caution in interpreting the results on inequalities is required. Specifically, preventable mortality can be considered a reliable proxy of incidence, and the observed inequalities in mortality for this group of causes are mainly influenced by public health policies and interventions. Conversely, treatable mortality can be interpreted as a proxy of lethality, representing the combination of incidence and survival. Thus, inequalities in treatable mortality should be interpreted bearing in mind that they represent an average of both inequalities in incidence (little influenced by care) and inequalities in survival (strongly influenced by care) and that these two components can sometimes have opposite trends.

Unfortunately, the short time span covered by our data did not allow us to analyze the trend of inequalities in avoidable mortality, although studies looking at trends in amenable mortality by socioeconomic group have been rare [38]. However, we can interpret the results, and especially the policy implications, in light of these limitations in the construct of indicators.

Furthermore, some people may have moved during the follow-up period, resulting in misclassification of residence at the time of death. Indeed, between 2012 and 2019, about 2–2.5% of the population annually changed their residence within Italy. However, 70–90% of these relocations took place within the same macro area of residence (e.g., from one region to another in the North-West) [39].

Lastly, the retrospective design of the cohort does not make it possible to update baseline information over the course of follow-up or to analyze other potential exposures and confounders not collected in the Census [17]. This could represent a limitation when studying the relationship between education level and mortality, especially for the younger age groups.

We would also like to mention a more conceptual issue intrinsic to the definition of avoidable mortality. Attributing an outcome to particular aspects of health care and health policy is problematic due to the multidimensional nature of most outcomes. Thus, because deaths from multiple causes are the final stage in a complex chain of events, some shaped by underlying social and economic factors, lifestyles, and previous use of preventive and curative health care, it is difficult to establish a direct association between treatable mortality and the efficiency of the healthcare system. Another potential problem in interpreting the results could be that the severity of diseases varies by sex and between socioeconomic groups. An excess mortality rate for less educated people compared to those who are more educated could be due to the fact that the health status of the former group is generally poorer. In that case, some of the differences in mortality may not be caused by deficiencies in the quality of health care the group has received but by the poorer case mix for less educated people [40].

## Conclusions

The present study, which analyzed inequalities in avoidable mortality, can contribute considerably to orienting equity-grounded health policies in Italy by suggesting priority areas of intervention for less educated people, who are more impacted by deficiencies in prevention and in treatment.

It has been demonstrated that higher health-care expenditure can reduce absolute inequalities in mortality, at least in the European context, in which most countries have universal health care or welfare systems, which to some degree guarantees equality of access to health care. In fact, although the effect of an increase in health care access would be equally strong in relative terms among both people with a low education level and those with high level, larger absolute effects are to be expected for the former group, as they have higher starting levels of treatable mortality compared to the latter.

The mortality inequalities in Italy represent a possible missed gain in health, suggesting the need to reassess priorities and define health targets. Forty-five years after the Italian National Health Service was instituted, the goal of health equity has not yet been fully achieved. The persistence of areas of inequality in health, with an extremely varied response at the regional level, suggests differences in prognosis correlated to the quality of care and draws attention to the need for greater coordination and for the development of action plans that are more systematic and effective [10].

In the light of the greater impact of avoidable mortality in southern Italy in absolute and in relative terms, further investment must be considered for this geographic area. A recent English study in fact demonstrated that investing in deprived geographic areas can lead to a significant reduction in inequality in avoidable mortality [41].

According to the WHO definition of health systems, whose primary aim is to promote, restore, and maintain health, inequalities in avoidable mortality must continue to be considered a strong indicator of possible critical issues in the primary prevention chain or in health care across social groups of the population, making them also a useful tool with which to evaluate public health policies [12].

### Electronic supplementary material

Below is the link to the electronic supplementary material.

Supplementary Material 1

Supplementary Material 2

## Data Availability

No datasets were generated or analysed during the current study.
