# Supplementary material for: Socioeconomic inequalities in avoidable mortality in Italy: results from a nationwide longitudinal cohort
Source: BMC Public Health. 2024 Mar 11;24:757. doi: 10.1186/s12889-024-18205-6 (PMC10929136; doi:10.1186/s12889-024-18205-6)
Supplement: Supplementary file 1 — Supplementary Material 1 [file 12889_2024_18205_MOESM1_ESM.docx]

**Supplementary Table 1. Mortality rate ratios (MRRs) for macro area of residence (reference category "North-West"), adjusted for age and education level, by group of causes and sex.**

| **GROUP OF CAUSES** | **MACRO AREA OF RESIDENCE** | **MALES** | | **FEMALES** | |
| --- | --- | --- | --- | --- | --- |
|  |  | **MRR** | **95%CI** | **MRR** | **95%CI** |
| **TOTAL** | North-West | 1 |  | 1 |  |
|  | North-East | 0.97 | 0.96-0.98 | 0.96 | 0.95-0.98 |
|  | Center | 1.00 | 0.99-1.02 | 1.01 | 1.00-1.03 |
|  | South and Islands | **1.11** | **1.10-1.13** | **1.11** | **1.09-1.12** |
| **PREVENTABLE** | North-West | 1 |  | 1 |  |
|  | North-East | 0.95 | 0.93-0.96 | 0.96 | 0.94-0.97 |
|  | Center | 0.99 | 0.97-1.00 | 1.02 | 1.01-1.04 |
|  | South and Islands | **1.08** | **1.07-1.09** | **1.04** | **1.02-1.05** |
| **TREATABLE** | North-West | 1 |  | 1 |  |
|  | North-East | 0.97 | 0.95-0.98 | 0.93 | 0.92-0.95 |
|  | Center | 1.07 | 1.05-1.08 | 1.01 | 0.99-1.03 |
|  | South and Islands | **1.21** | **1.19-1.23** | **1.18** | **1.17-1.20** |
| **NON-AVOIDABLE** | North-West | 1 |  | 1 |  |
|  | North-East | 1.01 | 0.99-1.02 | 0.99 | 0.98-1.01 |
|  | Center | 0.99 | 0.97-1.00 | 1.01 | 0.99-1.03 |
|  | South and Islands | **1.10** | **1.08-1.11** | **1.10** | **1.08-1.12** |
