# Supplementary material for: Socioeconomic inequalities in avoidable mortality in Italy: results from a nationwide longitudinal cohort
Source: BMC Public Health. 2024 Mar 11;24:757. doi: 10.1186/s12889-024-18205-6 (PMC10929136; doi:10.1186/s12889-024-18205-6)
Supplement: Supplementary file 2 — Supplementary Material 2 [file 12889_2024_18205_MOESM2_ESM.docx]

**Supplementary Table 2. Relative index of inequalities (RII) for education level by group of causes and age classes, adjusted for age and macro area of residence.**

| **Age group** | **Group of Causes** | **MALES** | | **FEMALES** | |
| --- | --- | --- | --- | --- | --- |
|  |  | **RII** | **95%CI** | **RII** | **95%CI** |
| **30-74** | **Preventable** | 2.61 | 2.56-2.66 | 1.73 | 1.69-1.76 |
|  | **Amenable** | 2.04 | 2.00-2.08 | 1.52 | 1.49-1.55 |
|  | **Non-avoidable** | 1.79 | 1.75-1.82 | 1.57 | 1.53-1.61 |
|  | **Total** | 2.18 | 2.14-2.22 | 1.60 | 1.57-1.63 |
| **30-59** | **Preventable** | 4.03 | 3.88-4.17 | 2.54 | 2.45-2.64 |
|  | **Amenable** | 3.09 | 2.96-3.22 | 1.76 | 1.70-1.83 |
|  | **Non-avoidable** | 2.87 | 2.75-3.00 | 2.25 | 2.15-2.35 |
|  | **Total** | 3.42 | 3.30-3.54 | 2.14 | 2.07-2.21 |
| **60-74** | **Preventable** | 2.24 | 2.19-2.29 | 1.49 | 1.45-1.53 |
|  | **Amenable** | 1.84 | 1.80-1.89 | 1.42 | 1.39-1.46 |
|  | **Non-avoidable** | 1.54 | 1.51-1.57 | 1.37 | 1.33-1.40 |
|  | **Total** | 1.89 | 1.86-1.93 | 1.42 | 1.39-1.45 |
